# Dietary Supplement Bioactives: When Rigorous Frameworks Meet Regulatory Uncertainty

**DOI:** 10.1016/j.advnut.2026.100635

**Published:** 2026-06-04

**Authors:** Kevin C Klatt

**Affiliations:** Department of Nutritional Sciences, Temerty Faculty of Medicine, University of Toronto, Toronto, ON, Canada

Since the establishment of the Recommended Dietary Allowances in 1941 [1], nutritional recommendations have focused on ensuring intakes of essential nutrients to prevent deficiency and maintain physiological function. The framework for making such recommendations was refined across the 20th century and resulted in a relatively intuitive process: *1*) define the essentiality of the nutrient; *2*) define the chemical compounds in food that can fulfill the nutritive function in the body; and *3*) derive intake recommendations from the dose-response relationships between nutritive compounds and an indicator of nutritional adequacy, accounting for differences in pharmacokinetics of different nutrient forms in the diet. Although complex in its implementation, this approach is reasonably straightforward, linking a relatively small number of compounds to essential nutrient functions. Such a framework has been relatively successful in reducing the risk of nutritional deficiencies but has limited applicability to the potentially large number of nonessential dietary bioactive components in the human diet for which there is a growing evidence base. New perspectives, published by Yates et al. [2] in 2021 and Novotony et al. [3] in this edition of the *Journal*, provide comprehensive perspectives on how to consider recommended intakes of dietary bioactives consumed via both foods and supplements.

Why do frameworks for developing recommended intakes for dietary bioactives differ? Unlike essential nutrients, nonessential dietary bioactives are comparatively unconstrained by linking intakes to an essential nutritive function in the body. Rather, intakes can be linked to a vast array of health indicators and outcomes, from improving structure and physiological functioning to modifying acute and chronic disease risk. The determinants of these health indicators and outcomes are multifactorial, and the absence of any single dietary bioactive compound or group of compounds does not result in an explicit health risk, unlike an essential nutrient would. As there is no essential nutritive function to link intakes to, the chemical diversity of bioactives to be considered is similarly unconstrained, theoretically inclusive of any individual constituent or mixture of constituents derived from the food metabolome, proteome, and/or metallome. Thus, authoritative bodies providing intake recommendations for dietary bioactives must make considerably more decisions about relevant health outcomes as well as which dietary bioactives to consider and how to group them, while determining the level of evidence necessary to make a recommendation, as not making one does not necessarily result in harm. This level of complexity renders it unsurprising that, to date, the National Academies has only established a recommended intake for 1 nonessential dietary bioactive: dietary fiber [4].

To address these many considerations, the framework by Yates et al. [2] recommends a 4-step process for developing recommended intakes from foods centering decisions around causal evidence linking specific bioactives to health indicators, inclusive of structure and function as well as chronic disease risk. This framework has since been applied by an expert panel to produce the first dietary bioactive intake recommendation for flavan-3-ols and cardiometabolic health [5], suggesting that at least some bioactives can meet a sufficient evidentiary standard. In this issue, Novotny et al. [3] adapt this framework for establishing recommended intakes to dietary supplement bioactives, not only using the same core elements of chemical characterization, safety, and efficacy but also addressing supplement-specific considerations, including supplement form, source, purity, dose, contaminants, and drug interactions. The framework ultimately outputs either summary statements of the evidence or explicit recommendations, depending on the certainty of evidence, and lends itself to an iterative process either informing new research and future reviews using the framework or continued postmarket surveillance and pharmacovigilance to continually reassess safety and monitoring.

The framework proposed by Novotny et al. [3] is undoubtedly academically rigorous. However, whether it will produce recommendations that result in meaningful clinical or regulatory impact is uncertain. Dietary supplements exist in a regulatory landscape that was defined over 3 decades ago by the Dietary Supplement Health and Education Act (DSHEA) and the Nutrition Labeling and Education Act (NLEA) of 1990, laying the statutory foundation for structure/function and authorized health claims for dietary supplements. DSHEA also defined the postmarket regulatory approach to dietary supplements, limiting the need to demonstrate premarket efficacy and safety (with the exception of new dietary ingredient notifications). Recommendations produced by the framework by Novotny et al. [3] will enter this regulatory landscape and are likely to be caught between evidentiary thresholds and progress limited by a circular incentive structure in which no stakeholder is clearly positioned to bridge evidentiary gaps. The framework’s graded evidence approach identifies safety-bounded, efficacious recommendations that constitute regulatory overkill for substantiating the structure-function claims that are most commonly made for supplements, limiting the need for such a framework to support these health links. The more central question is whether the framework will be used by professional and scientific bodies to produce broad clinical recommendations and establish significant scientific agreement (SSA) needed to support authorized health claims stating that dietary supplement bioactives reduce the risk of a relevant health outcome. On this issue, the framework lays out that a moderate certainty of evidence is needed to make recommendations, but this is likely to conflict with the high level of consistent evidence required by the NLEA to meet the SSA standard. The framework also allows for the use of surrogate end points to guide recommendations, yet major medical societies typically accept this standard only for dietary intake recommendations and not supplements (eg professional guidelines focused on preventing chronic cardiometabolic disease recommend consuming high fiber diets but typically do not recommend fiber supplements). Whether industry will be incentivized to fund high certainty, RCTs with disease end points to clear the evidentiary standard needed for recommendations by professional medical groups and to generate SSA remains uncertain. At present, there are few such well-designed, industry-funded trials to point to, with the exception of the recent Cocoa Supplementation and Multivitamin Outcomes Study (COSMOS) trial [6] testing the impact of cocoa extract supplement containing 500 mg cocoa flavanols with 80 mg (-)-epicatechin per day on cardiovascular disease risk. Even if such investment by the industry occurs, concerns about limited postmarket pharmacovigilance and surveillance systems for supplements, as well as issues of purity and contamination, remain and diminish many clinicians’ enthusiasm for recommending supplements.

Ultimately, the framework proposed by Novotny et al. [3] establishes a rigorous approach to evaluate the evidence for dietary supplement bioactives that lands on the regulatory architecture built by DSHEA, which does not require such a framework. In a >100-billion dollar marketplace where many consumers, and increasingly federal health regulators, believe in the efficacy of dietary supplements to reduce disease risk, and structure-function claims can imply efficacy (eg cocoa flavanols support circulation and promote heart health), one must wonder whether industry will ever be truly incentivized to invest in large-scale trials that feed into rigorous recommendation frameworks.

## Funding

The author reported no funding received for this editorial.

## Declaration of generative AI and AI-assisted technologies in the writing process

The author(s) used Claude (Anthropic, claude.ai, Claude Sonnet 4.6) in order to review spelling, grammar, and syntax of the final draft prior to submission. After using this tool/service, the author reviewed and edited the content and takes full responsibility for the content of the publication.

## Conflict of interest

KCK has no direct conflicts of interest relevant to this Editorial topic. Perceived conflicts of interest may include KCK’s previous funding from stakeholders in the supplement industry (Balchem) for research related to dietary choline supplementation during pregnancy.
